# A Robust and Adaptive Complementary Kalman Filter Based on Mahalanobis Distance for Ultra Wideband/Inertial Measurement Unit Fusion Positioning

**DOI:** 10.3390/s18103435

**Published:** 2018-10-12

**Authors:** Xin Li, Yan Wang, Kourosh Khoshelham

**Affiliations:** 1 School of Computer Science and Technology, China University of Mining and Technology, Xuzhou 221116, China; 2 Department of Infrastructure Engineering of the University of Melbourne, Melbourne 3010, Australia; k.khoshelham@unimelb.edu.au

**Keywords:** UWB/IMU fusion, adaptively-robust filter, complementary Kalman filter, Mahalanobis distance

## Abstract

Ultra wideband (UWB) has been a popular technology for indoor positioning due to its high accuracy. However, in many indoor application scenarios UWB measurements are influenced by outliers under non-line of sight (NLOS) conditions. To detect and eliminate outlying UWB observations, we propose a UWB/Inertial Measurement Unit (UWB/IMU) fusion filter based on a Complementary Kalman Filter to track the errors of position, velocity and direction. By using the least squares method, the positioning residual of the UWB observation is calculated, the robustness factor of the observation is determined, and an observation weight is dynamically set. When the robustness factor does not exceed a pre-defined threshold, the observed value is considered trusted, and adaptive filtering is used to track the system state, while the abnormity of system state, which might be caused by IMU data exceptions or unreasonable noise settings, is detected by using Mahalanobis distance from the observation to the prior distribution. When the robustness factor exceeds the threshold, the observed value is considered abnormal, and robust filtering is used, whereby the impact of UWB data exceptions on the positioning results is reduced by exploiting Mahalanobis distance. Experimental results show that the observation error can be effectively estimated, and the proposed algorithm can achieve an improved positioning accuracy when affected by outlying system states of different quantity as well as outlying observations of different proportion.

## 1. Introduction

Indoor positioning technology is important in a variety of applications, ranging from supermarket shopping to drone positioning and hospital patient tracking [1,2,3]. The ultra wideband (UWB) positioning technology has been particularly popular since it can achieve decimeter-level positioning accuracy under line of sight (LOS) conditions. However, in many practical scenarios such as warehouse robot positioning and emergency response, UWB signal attenuation and even signal loss occurs due to obstruction by personnel and cargo and multi-path effects, resulting in a sharp drop in UWB positioning accuracy [4,5]. The UWB/IMU fusion is an effective way to achieve high-precision positioning under non-line of sight (NLOS) conditions. However, long time positioning cannot be maintained due to the susceptibility of IMU data to integral accumulation errors. In addition, when the motion state of the carrier changes drastically, such as during bumping and braking, the IMU data is easily disturbed by abnormal measurements. Therefore, improving positioning accuracy of UWB data in NLOS conditions and when IMU data is affected by abnormal values is a hot research topic.

Extensive research has been done on UWB/IMU fusion for positioning applications. Sczyslo et al. [6,7] used loose coupling and tracked the pedestrian movement based on an Extended Kalman Filter (EKF). Ascher et al. [8] presented a tightly coupled UWB/IMU system for indoor applications, with IMUs mounted on pedestrians and moving cars. Blanco et al. [9] used a particle filter algorithm to fuse UWB, IMU and odometer data, achieving improved positioning performance under NLOS conditions. Xu et al. [10] developed a new approach using least squares support vector machine and H∞ filter for IMU/wireless sensor network (WSN) integration and achieved a reduction of positioning error by 14.8% compared with the UWB-only model. Wang et al. [11] designed a tightly-coupled Global Positioning System (GPS)/UWB/IMU integrated system, achieving high positioning accuracy in outdoor environments. Benini et al. [12] proposed a positioning method based on the fusion of vision, IMU and UWB onboard on a flying drone, achieving two-dimensional positioning accuracy of 10 cm.

In the existing UWB/IMU fusion methods, the effect of outliers is either ignored [13,14,15], or it is mitigated by using zero velocity update (ZUPT) or pedestrian dead reckoning (PDR), which are applicable to pedestrians only and cannot be used for moving platforms such as robots or forklifts. Other methods use additional sensors such as vision, GPS and odometers to compensate for the outliers in UWB/IMU data, which makes the positioning system less affordable and more computationally expensive.

In this paper, we propose a method to minimize the effect of outliers in UWB/IMU fusion. In the proposed method, the state error is estimated by using a Complementary Kalman Filter (CKF) [16,17,18], and the error of position, velocity and direction as well as the bias of accelerometer and gyroscope are contained in the state parameters. The observations are obtained from the difference between the UWB ranging and the distance from the beacon to the position obtained by IMU integration at 2 Hz. Each time the system state is updated, the position, velocity and direction errors contained in it are directly fed back to the navigation equation to calculate the result of the error correction, and the bias of the acceleration and the gyroscope are used to correct the original value of the accelerometer and the gyroscope, respectively. The advantage of this approach is that it can be used for both vehicles and pedestrian applications. In addition, the state error rather than the state itself is stored by the algorithm, so that a smaller value is used for approximation when linearizing the system, resulting in relatively more accurate results. Since the standard CKF algorithm is sensitive to non-Gaussian noise [19,20], a Robust CKF is proposed to adjust the observation covariance thereby reducing the influence of non-Gaussian noise on positioning accuracy.

The influence of abnormal observations is also eliminated by using the Robust CKF algorithm, improving the positioning accuracy. However, only the prior information is used by the Robust CKF algorithm to judge the reliability of UWB observations which will be invalid when there is a problem in the system state. If the system state deviates from the true trajectory, the following correct observations may be identified as outliers, making it difficult to drag back the system state to the real trajectory by the constraint of the UWB measurements. Due to the bias of the system state, the abnormal observations are misjudged as correct ones, while the correct observations present rather small confidence since they deviate from the wrong system state. Compared with the Standard CKF algorithm, it is more difficult for the Robust CKF algorithm to recover from the wrong state to the correct one.

In order to suppress the influence of abnormal system state and observations on positioning accuracy, an Adaptive-Robust filtering method based on Mahalanobis distance and robustness factor is designed in this paper. The positioning residual is optimized by UWB to determine the robustness factor and identify the abnormal UWB observations. When the observations are credible, the Adaptive-Robust filtering algorithm is executed; when the observations are abnormal, only the robust part of the Adaptive-Robust filtering algorithm is executed.

The remainder of the paper is organized as follows: in Section 2, the UWB/IMU fusion algorithm based on CKF is discussed, and the motion model and observation model of the algorithm are introduced. The principle of the Adaptive-Robust algorithm based on Mahalanobis distance is presented in Section 3, including a description of the calculation of the robustness factor of the observation. Several experiments are conducted and the results are analyzed in Section 4. Finally, conclusions are drawn in Section 5.

## 2. The UWB/IMU Fusion Filter

**Figure 1:**
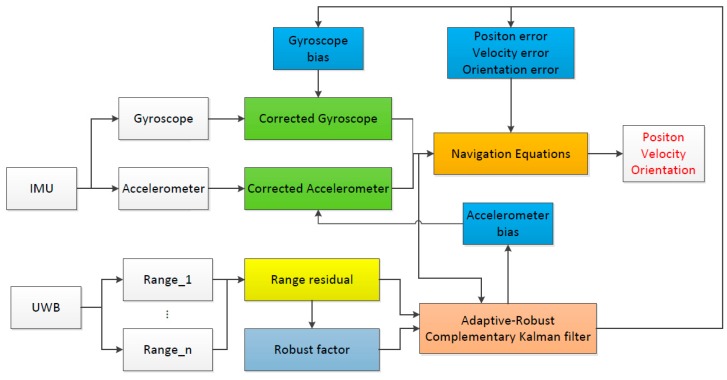
Flowchart of the proposed Adaptive-Robust CKF.

An overview of the proposed UWB/IMU fusion method is shown in Figure 1. As illustrated, the method is based on the CKF algorithm, and the Adaptive-Robust filtering is conducted on the abnormal system state or abnormal observations according to the robustness factor of the observations. Then, the state error obtained by filtering is fed back to the Navigation Equations to calculate the position, velocity and direction. The core of the algorithm is divided into four parts and discussed in this section.

### 2.1. IMU Navigation Equations

Following the method in [21], the state in the CKF algorithm is defined as: $X=[\delta p^{n}\;\delta v^{n}\;\varepsilon \;b_{g}b_{a}$], where $\delta p^{n}$ is the position error, $\delta v^{n}$ is the velocity error, ε is direction error, $b_{g}$ is the gyroscope bias, and $b_{a}$ is the acceleration bias. X is a 15-dimensional vector. The first three navigation state vectors are defined in navigation frame (*n*-frame), and the last two bias vectors are in body frame (*b*-frame).

By integrating the gyroscope and acceleration data, the position, velocity and direction data in n-frame can be obtained. The IMU navigation equation in continuous-time state is defined as:

(1) $${\dot{p}}^{n}=v^{n}$$

(2) $${\dot{v}}^{n}=C_{b}^{n}f^{b}+g^{n}$$

(3) $${\dot{C}}_{b}^{n}=C_{b}^{n}[\omega^{b}\times ]$$

$C_{b}^{n}$ represents the transformation from *b*-frame to *n*-frame. ${\dot{p}}^{n},{\dot{v}}^{n},{\dot{C}}_{b}^{n}$ represent the first derivative of position, velocity and attitude, respectively. $g^{n}$ is the gravity vector under *n*-frame, $f^{b}$ is the acceleration vector under *b*-frame, and $\omega^{b}=\left[\omega_{x}^{b},\omega_{y}^{b},\omega_{z}^{b}\right]$ is the angular velocity in *b*-frame. $\left[\omega^{b}\times \right]$ is the skew-symmetric matrix of angular velocity, defined as follows:

For the acceleration and the gyroscope observations at moment *k*, their biases $b_{a}$ and $b_{g}$, which are estimated as the state parameters of the CKF algorithm, must be firstly removed as follows:

(4) $$[\omega^{b}\times ]=\left[\begin{array}{ccc} 0 & -\omega_{z}^{b} & \omega_{y}^{b}\\ \omega_{z}^{b} & 0 & -\omega_{x}^{b}\\ -\omega_{y}^{b} & \omega_{x}^{b} & 0 \end{array}\right]$$

(5) $${\hat{f}}_{k}^{b}={\tilde{f}}_{k}^{b}-b_{a}$$

(6) $${\hat{\omega }}_{k}^{b}={\tilde{\omega }}_{k}^{b}-b_{g}$$

${\tilde{f}}_{k}^{b}$ and ${\tilde{\omega }}_{k}^{b}$ represent the observations of the original acceleration and angular velocity, respectively, and ${\hat{f}}_{k}^{b}$ and ${\hat{\omega }}_{k}^{b}$ are the values after the biases are compensated. The acceleration transformation from moment k to moment (*k* + 1) is:

(7) $${\hat{f}}_{k+1}^{n}=C_{b}^{n}\left({\hat{f}}_{k}^{b}+0.5\left({\hat{\omega }}_{k}^{b}d_{t}⨂{\hat{f}}_{k}^{b}\right)\right)-g^{n}$$

⨂ denotes vector cross product, representing the rotation correction on the acceleration by the angular velocity change.

The velocity transformation from moment *k* to moment (*k* + 1) is:

(8) $$v_{k+1}^{n}=v_{k}^{n}+{\hat{f}}_{k+1}^{n}d_{t}-\delta v_{k}^{n}$$

$\delta v_{k}^{n}$ represents the velocity error at moment *k*, which is estimated as the state parameter of the CKF algorithm.

The position transformation from moment k to moment (*k* + 1) is:

(9) $$p_{k+1}^{n}=p_{k}^{n}+0.5(v_{k}^{n}+v_{k+1}^{n})d_{t}-\delta p_{k}^{n}$$

$\delta p_{k}^{n}$ represents the position error at moment *k*, which is estimated as the state parameter of the CKF algorithm.

The attitude transformation from moment *k* to moment (*k* + 1) is:

(10) $$C_{b,k+1}^{n}{}^{\prime }=\left(I-\left[\varepsilon \times \right]\right)C_{b,k}^{n}$$

(11) $$C_{b,k+1}^{n}=C_{b,k+1}^{n}{}^{\prime }\left(I+\left[{\hat{\omega }}_{k}^{b}d_{t}\times \right]\right)$$

Firstly, the correction of direction error is conducted on the attitude change matrix $C_{b,k}^{n}$ at moment *k*, and $C_{b,k+1}^{n}{}^{\prime }$ is obtained, wherein the direction error *ε* is cyclically calculated by the CKF algorithm. Then, the compensated rotation from moment k to moment (*k* + 1) is conducted on $C_{b,k+1}^{n}{}^{\prime }$, and $C_{b,k+1}^{n}$, the rotation matrix at moment (*k* + 1) is obtained. In order to improve the system stability, the value of $C_{b,k+1}^{n}$ should be periodically normalized, otherwise the matrix $C_{b,k+1}^{n}$ might become singular.

### 2.2. State Transformation Model

In order to track the five state parameters in the state model of the CKF algorithm, the state transformation model must be derived. The differential equation of the system dynamic model under continuous-time is defined as follows:

(12) $$\dot{X}=FX+W$$

where F is the state transformation matrix of the system, and W is the system noise. Since the inputs of the IMU and UWB are discretized data, Equation (12) is discretized as:

(13) $$X_{k+1}=\emptyset_{k}X_{k}+W_{k}$$

(14) $$\emptyset_{k}=e^{F_{k}dt}$$

In order to determine $F_{k}$, the transformation formula of state $X=[\delta p^{n}\;\delta v^{n}\;\varepsilon \;b_{g}\;b_{a}$] must be derived, which consists of the following steps.

(1) Equation of acceleration bias and gyroscope bias

The measurement equations for acceleration and gyroscope are as follows:

(15) $${\tilde{f}}^{b}=f^{b}+b_{a}+n_{a}$$

(16) $${\tilde{\omega }}^{b}=\omega^{b}+b_{g}+n_{g}$$

where ${\tilde{f}}^{b}$ and ${\tilde{\omega }}^{b}$ are the measurements of the acceleration and the angular velocity respectively; $f^{b}$ and $\omega^{b}$ are the true values of the acceleration and the angular velocity respectively. n is the measurement noise which obeys the Gaussian distribution, and its covariance is defined as $N_{a}$ and $N_{g}$, respectively for acceleration and angular velocity. b is the drift bias, which is a time-dependent first-order Markov process, defined for acceleration and angular velocity as:

(17) $${\dot{b}}_{a}=-t_{a}{}^{-1}b_{a}+\mu_{a}$$

(18) $${\dot{b}}_{g}=-t_{g}{}^{-1}\;b_{g}+\mu_{g}$$

μ is the offset noise which obeys the Gaussian distribution. The covariance of the acceleration and the gyroscope is defined as $U_{a}$ and $U_{g}$, respectively.

(2) Equation of direction error

The direction error *ε* is caused by the gyroscope bias and is defined as:

(19) $$\dot{\varepsilon }=C_{b}^{n}\delta \omega^{b}$$

It indicates the transformation of $\delta \omega^{b}$, the measured gyroscope bias, from *b*-frame to *n*-frame. $\delta \omega^{b}$ is caused by the drift bias and noise of the gyroscope.

(20) $$\delta \omega^{b}=b_{g}+n_{g}$$

(3) Equation of velocity error and position error

The velocity error is caused by the acceleration error. Since the direction error can result in acceleration error, which will further result in velocity error, the velocity error is defined as:

(21) $$\delta {\dot{v}}^{n}=\left[f^{n}\times \right]\varepsilon +C_{b}^{n}\delta f^{b}$$

where $\left[f^{n}\times \right]\varepsilon$ represents the influence of the direction error on the acceleration, and $\delta f^{b}$ is the measurement error caused by the drift bias and noise of the acceleration, defined as:

(22) $$\delta f^{b}=b_{a}+n_{a}$$

The position error is defined as:

(23) $$\delta {\dot{p}}^{n}=\delta v^{n}$$

Based on the state transformation equation of the five parameters in $X=[\delta p^{n}\;\delta v^{n}\;\varepsilon \;b_{g}\;b_{a}$], the state transformation matrix F of the system is derived as:

(24) $$F=\left[\begin{array}{c} 0\\ 0\\ 0\\ 0\\ 0 \end{array}\begin{array}{c} I\\ 0\\ 0\\ 0\\ 0 \end{array}\begin{array}{c} 0\\ \left[f^{n}\times \right]\\ 0\\ 0\\ 0 \end{array}\begin{array}{c} 0\\ 0\\ -C_{b}^{n}\\ diag\left(-t_{g}{}^{-1}\right)\\ 0 \end{array}\begin{array}{c} 0\\ C_{b}^{n}\\ 0\\ 0\\ diag\left(-t_{a}{}^{-1}\right) \end{array}\right]$$

where 0 and I represent a 3 × 3 null matrix and a 3 × 3 identity matrix, respectively. The system noise W is:

(25) $$W=\left[\begin{array}{c} 0\\ C_{b}^{n}n_{a}\\ -C_{b}^{n}n_{g}\\ \mu_{a}\\ \mu_{g} \end{array}\right]$$

The covariance matrix Q of the system noise W is:

(26) $$Q=\left[\begin{array}{c} N_{a}\\ 0\\ 0\\ 0 \end{array}\begin{array}{c} 0\\ N_{g}\\ 0\\ 0 \end{array}\begin{array}{c} 0\\ 0\\ U_{a}\\ 0 \end{array}\begin{array}{c} 0\\ 0\\ 0\\ U_{g} \end{array}\right]$$

where N and U represent 3 × 3 diagonal matrices, and the noise transformation matrix is defined as:

(27) $$G=\left[\begin{array}{c} 0\\ C_{b}^{n}\\ 0\\ 0\\ 0 \end{array}\begin{array}{c} 0\\ 0\\ -C_{b}^{n}\\ 0\\ 0 \end{array}\begin{array}{c} 0\\ 0\\ 0\\ I\\ 0 \end{array}\begin{array}{c} 0\\ 0\\ 0\\ 0\\ I \end{array}\right]$$

We discretize the noise covariance matrix Q to get $Q_{k}$ as follows:

(28) $$Q_{k}=\frac{1}{2}\left(\emptyset_{k}GQG^{\prime }+GQG^{\prime }\emptyset_{k}{}^{\prime }\right)$$

By now, $\emptyset_{k}$ and $Q_{k}$ in the CKF algorithm has been defined. $Z_{k}$,$H_{k},R_{k}$ and other matrices will be defined in Section 2.3.

### 2.3. Observation Model

Given the known coordinates of n beacons denoted as $S_{i}=\left(S_{x,i},S_{y,i},S_{z,i}\right)$, i∈(1,n), the observation function is defined as:

(29) $$h\left(\delta {\hat{p}}_{k}^{n}\right)=\left[\begin{array}{c} \left\|S_{1}-\left(\delta {\hat{p}}_{k}^{n}+{\hat{p}}_{ins,k}\right)\|-\|S_{1}-{\hat{p}}_{ins,k}\right\|\\ \vdots \\ \left\|S_{n}-\left(\delta {\hat{p}}_{k}^{n}+{\hat{p}}_{ins,k}\right)\|-\|S_{n}-{\hat{p}}_{ins,k}\right\| \end{array}\right]$$

where ${\hat{p}}_{ins,k}=\left({\hat{x}}_{k},{\hat{y}}_{k},{\hat{z}}_{k}\right)$ is the position coordinate calculated by the IMU; $\delta {\hat{p}}_{k}^{n}=\left(\delta {\hat{x}}_{k},\delta {\hat{y}}_{k},\delta {\hat{z}}_{k}\right)$ is the priori position error calculated by the state transformation equation; ‖.‖ represents the Euclidean distance. Equation (29) represents the difference between two ranging values: the first ranging value is from the beacon to the IMU integration position including the position error; the second ranging value is from the beacon to the IMU integration position excluding the position error. The Jacobian matrix of the observation equation is defined as:

(30) $$\begin{array}{l} H_{k}=\frac{\delta (h(\delta {\hat{p}}_{k}^{n}))}{\delta (\delta {\hat{p}}_{k}^{n})}\\ =[\begin{array}{cccc} \frac{S_{x,1}-({\hat{x}}_{k}+\delta {\hat{x}}_{k})}{\left\|S_{1}-(\delta {\hat{p}}_{k}^{n}+{\hat{p}}_{ins,k})\right\|} & \frac{S_{y,1}-({\hat{y}}_{k}+\delta {\hat{y}}_{k})}{\left\|S_{1}-(\delta {\hat{p}}_{k}^{n}+{\hat{p}}_{ins,k})\right\|} & \frac{S_{z,1}-({\hat{z}}_{k}+\delta {\hat{z}}_{k})}{\left\|S_{1}-(\delta {\hat{p}}_{k}^{n}+{\hat{p}}_{ins,k})\right\|} & 0_{1\times 12}\\ \vdots & \vdots & \vdots & \vdots \\ \frac{S_{x,n}-({\hat{x}}_{k}+\delta {\hat{x}}_{k})}{\left\|S_{n}-(\delta {\hat{p}}_{k}^{n}+{\hat{p}}_{ins,k})\right\|} & \frac{S_{y,n}-({\hat{y}}_{k}+\delta {\hat{y}}_{k})}{\left\|S_{n}-(\delta {\hat{p}}_{k}^{n}+{\hat{p}}_{ins,k})\right\|} & \frac{S_{z,n}-({\hat{z}}_{k}+\delta {\hat{z}}_{k})}{\left\|S_{n}-(\delta {\hat{p}}_{k}^{n}+{\hat{p}}_{ins,k})\right\|} & 0_{1\times 12} \end{array}] \end{array}$$

The observation is defined as:

(31) $$Z_{k,i}=r_{k,i}-\|S_{i}-{\hat{p}}_{ins,k}\|$$

where $r_{k}$ is the ranging data of UWB. Equation (31) represents the difference between the UWB ranging and the distance from the beacon to the position obtained by IMU integration.

The observation equation is defined as:

(32) $$Z_{k}=H_{k}X_{k}+V_{k}$$

where $V_{k}$ is the measurement noise matrix, and $V_{k}\sim N\left(0,R_{k}\right)$. $R_{k}$ is the covariance matrix.

## 3. Adaptive-Robust Filtering Strategy

The accuracy of UWB sensor observations is affected by ambient temperature, power supply stability, fixed obstacles, and even people or objects moving in the environment. Therefore, the confidence of UWB observations must be estimated. A calculation method based on robustness factor is designed in this paper. The robustness factor is used to adjust the effect of the observation error on the system. The observation error here refers to the error within a certain range, and the observation with particularly large error is defined as abnormal value (outliers) which is processed by the method based on Mahalanobis distance.

### 3.1. Calculation of Robustness Factor

**Figure 2:**
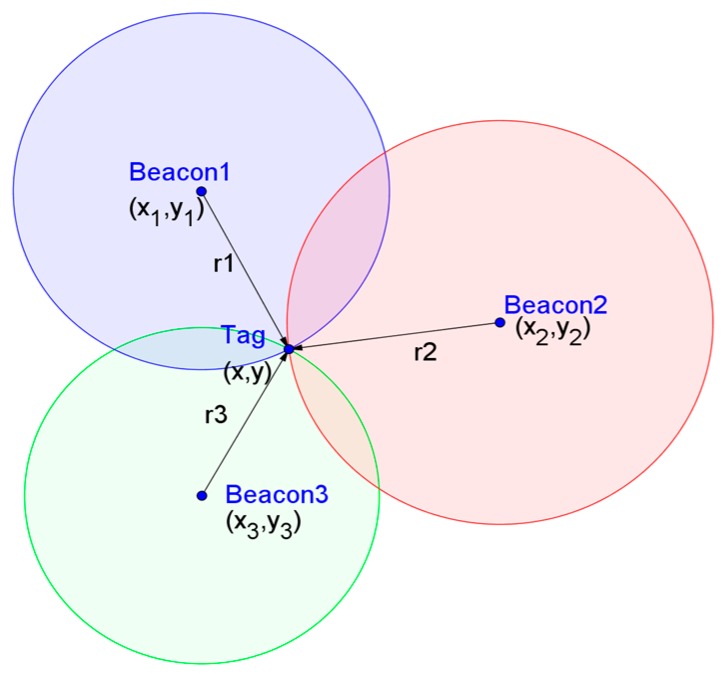
Position estimation by trilateration without measurement error.

To simplify the description, assume that three beacons are adopted. In the planar positioning, the *i*th UWB beacon is denoted as ${\mathrm{Beacon}}_{i}$, and the corresponding coordinates are $\left(x_{i},y_{i}\right)$. The UWB tag used for positioning is denoted as Tag, and its coordinates are (*x*, *y*). The true distance between the Tag and ${\mathrm{Beacon}}_{i}$ is denoted as $r_{i}$, and its corresponding measurement is denoted as $r_{i}^{\prime }$. As shown in Figure 2, ideally, $r_{i}^{\prime }=r_{i}$, the three circles will intersect at a unique point, and its coordinates indicate the position of the tag under the current observation data. To solve this intersection point, an error function is defined and the coordinate of Tag is obtained by minimizing the error function. A feasible error function is:

(33) $$E\left(x,y\right)=\overset{n}{\underset{i}{\sum }}\left|\left(\sqrt{\left({\left(x-x_{i}\right)}^{2}+{\left(y-y_{i}\right)}^{2}\right)}-r_{i}^{\prime }\right)\right|$$

In Equation (33), |.| represents absolute value function. The estimated coordinates of the tag $\left(x^{\prime },y^{\prime }\right)$, can be obtained by minimizing E(x,y):

(34) $$\left(x^{\prime },y^{\prime }\right)=argmin_{\left(x,y\right)}E\left(x,y\right)$$

Ideally, the minimum value of E(x,y) is zero. However, in practice, the measurements contain error. Assume that $r_{i}$ still represents the true distance, and its corresponding error is denoted as $\Delta r_{i}$. At this time, the measurement of the corresponding ${\mathrm{Beacon}}_{i}$ is $r_{i}^{\prime }=r_{i}+\Delta r_{i}$.

**Figure 3:**
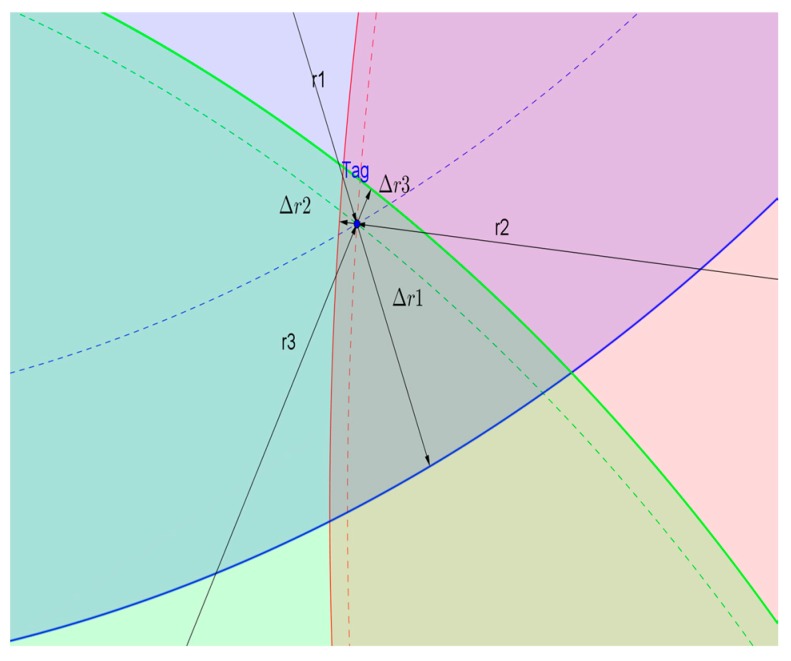
Position estimation by trilateration with measurement error.

The partial trilateration diagram is shown in Figure 3, showing the intersection of the circles corresponding to the measurements of the three Beacons around the Tag. Obviously, when the measurements contain error, the three circles will intersect each other rather than intersect at one point. In this case, $\left(x^{\prime },y^{\prime }\right)$, the estimated value of the Tag coordinate, is still obtained by minimizing E(x,y).

The robustness factor of the UWB data is defined as:

(35) $$C_{ui}=\{\begin{array}{ll} e^{\left|\Delta r_{i}\right|} & C_{ui}<C_{u\_max}\\ C_{u\_max} & C_{ui}\geq C_{u\_max} \end{array}$$

**Figure 4:**
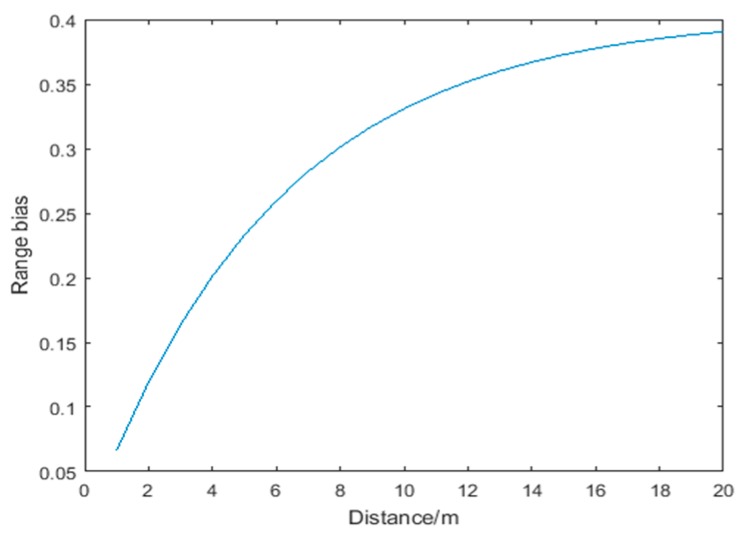
Ranging error when distance *d* is within 20 m.

$e^{\left|\Delta r_{i}\right|}\in [1,+\infty$], i∈[1,n]. The larger $\left|\Delta r_{i}\right|$ is, the less trustworthy the data is. $C_{u\_max}$ has two functions: firstly, the value of $C_{ui}$ must be limited within a certain range, otherwise it will lead to matrix singularity when $C_{ui}$ is used to modify $R_{k}$; secondly, it is used as the threshold of the observation error, and the observation error larger than it is treated as outliers and processed by the method based on the Mahalanobis distance. The value of $C_{u\_max}$ is determined by the UWB ranging error within the environment. Generally, the ranging error will increase with increasing distance. Following [9], the UWB ranging error is defined as:

(36) $$f\left(d\right)=0.4\left(1.10-e^{-0.17d}\right)$$

where f(d) represents the ranging error at range d. Figure 4 shows the error distribution when d is within 20 m. Assume that the ranging error obeys the Gaussian distribution, that is, 97% of the ranging errors are within f(d)±3σ, and the value of the ranging error greater than f(d)+3σ is regarded as an outlier. Thus, $C_{u\_max}$ is defined as:

(37) $$C_{u\_max}=e^{f\left(d\right)+3\sigma }$$

**Figure 5:**
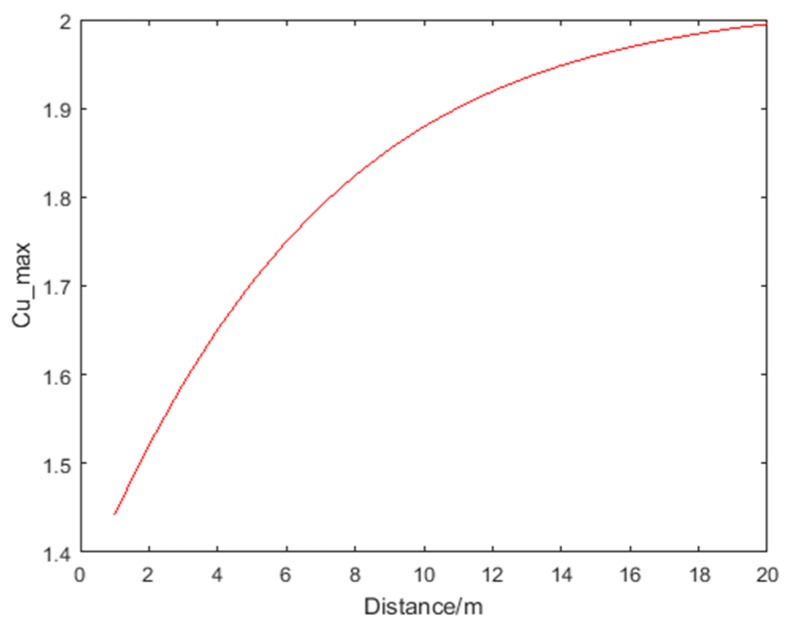
$C_{u\_max}$ for *d* within 20 m and *σ* = 0.1 m.

Figure 5 shows the $C_{u\_max}$ for distance d within 20 m. In the experiment environment described in Section 4, *σ* is set to 0.1 m according to UWB positioning tests.

Finally, $R_{k}$ is defined as:

(38) $$R_{k}=\left[\begin{array}{cccc} \sigma_{s1}^{2}C_{u1} & 0 & 0 & 0\\ 0 & \sigma_{s2}^{2}C_{u2} & 0 & 0\\ 0 & 0 & \sigma_{s3}^{2}C_{u3} & 0\\ 0 & 0 & 0 & \sigma_{s4}^{2}C_{u4} \end{array}\right]$$

$\sigma_{si}^{2}$ represents the covariance of the ranging from the *i*th UWB beacon to the tag. If the number of range measurements is less than 3, e.g., due to occlusion, an effective residual value cannot be obtained, in which case we set $C_{u}$ to 1.

### 3.2. Adaptive-Robust Filtering Based on Mahalanobis Distance

In general, the noise of the UWB measurement under LOS (Line of Sight) condition obeys the Gaussian distribution, and the observation covariance can be well adjusted by the robustness factor mentioned above, so that the quality of the ranging value can be quantitatively evaluated. However, in the NLOS environment, due to the influence of refraction, obstacles and other factors, the noise model is often difficult to estimate and abnormal observations might appear. To solve this problem, the Mahalanobis distance is used to determine the observation covariance.

Suppose that the noise of the system observation $Z_{k,i}$ obeys the Gaussian distribution, that is, the observation $Z_{k,i}$ obeys the Gaussian distribution with the mean of $H_{k,i}X_{k}$ and the variance of $H_{X_{k},i}P_{k}^{-}H_{X_{k},i}^{T}-R_{k,i}$. The subscript i here is associated with the specific beacon, and $P_{k}^{-}$ is the a priori covariance of the system. $\gamma_{k,i}$, the square of the Mahalanobis Distance from $Z_{k,i}$ to $H_{k,i}X_{k}$ obeys the $\chi^{2}$ distribution [22]:

(39) $$\gamma_{k,i}={\left(Z_{k,i}-H_{k,i}X_{k}\right)}^{T}{\left(H_{k,i}P_{k}^{-}H_{k,i}^{T}+R_{k,i}\right)}^{-1}\left(Z_{k,i}-H_{k,i}X_{k}\right)\sim \chi_{1}^{2}$$

where $\chi_{1}^{2}$ represents a $\chi^{2}$ distribution with the degree of freedom of 1. For the significance level α, we have:

(40) $$\mathrm{Pr}\left(\gamma_{k,i}<\chi_{1,\alpha }^{2}\right)=1-\alpha$$

where Pr() is the probability of a random event, and $\chi_{1,\alpha }^{2}$ is the α-quantile of the $\chi^{2}$ distribution with the degree of freedom of 1. An observation that does not pass this test is considered outlier and its covariance is increased to weaken its effect on the posteriori estimation. The new matrix of the observation covariance can be updated according to the following equation:

(41) $$R_{k,i}^{\prime }=\{\begin{array}{cc} R_{ki} & \gamma_{k,i}<\chi_{1,\alpha }^{2}\\ \left(\frac{\gamma_{k,i}}{\chi_{1,\alpha }^{2}}\right)\times R_{k,i} & \gamma_{k,i}\geq \chi_{1,\alpha }^{2} \end{array}$$

where $\frac{\gamma_{k,i}}{\chi_{1,\alpha }^{2}}$ represents the ratio of the Mahalanobis distance to the threshold at the current observation. In this paper, the significance level α is set to 0.001, and the corresponding value of $\chi_{1,\alpha }^{2}$ is 6.2 according to the Chi-Square Distribution Table.

On the other hand, if the observations are correct while the system state is abnormal, the method based on the Mahalanobis distance can also be used to correct the state. An abnormal system state is caused by two reasons: one is the error introduced to the system model due to sudden variation of the state or some unknown biases; the other is the error caused by the incorrect knowledge of the statistics of the process or measurement noises, such as the introduction of unreasonable covariance matrix or an assumed Gaussian distribution perturbed by other distributions. Once an abnormal system state is detected, a fading factor is introduced to inflate the covariance matrix of the state prediction so as to make the filter adaptive. Updating can be conducted according to the following equation:

(42) $$P_{k}^{-\prime }=\{\begin{array}{cc} P_{k}^{-} & \gamma_{k,i}<\chi_{1,\alpha }^{2}\\ \left(\frac{\gamma_{k,i}}{\chi_{1,\alpha }^{2}}\right)\times P_{k}^{-} & \gamma_{k,i}\geq \chi_{1,\alpha }^{2} \end{array}$$

It should be noted that when the correct observation is used to correct the system error, a certain time delay will be introduced, that is, since the occurrence of the abnormal system state, the state error cannot be corrected until the next correct observation comes in. This is slightly different from the abnormal observations that can be corrected in real time. To suppress the effects of the delayed correction, Rauch-Tung-Striebel (RTS) smoothing can be adopted to reverse-process the data from the occurrence of the abnormal system state to the next time the correct observation is received. The algorithm of the proposed Adaptive-Robust CKF is as follows Algorithm 1.
**Algorithm 1.** ARCKFFiltering: for *k* = 1, 2,… *1.*  *State prediction* *2.*  **For**
*each measurement* *3.*    **if** ($C_{ui}$ < $C_{u\_max}$) *4.*      **while**
$\gamma_{k,i}\geq \chi_{1,\alpha }^{2}$ *5.*       $P_{k}=\left(\frac{\gamma_{k,i}}{\chi_{1,\alpha }^{2}}\right)\times P_{k}$ *6.*    **else** *7.*      **while**
$\gamma_{k,i}\geq \chi_{1,\alpha }^{2}$ *8.*       $R_{k,i}=$
$\left(\frac{\gamma_{k,i}}{\chi_{1,\alpha }^{2}}\right)\times R_{k,i}$ *9.*    **end** *10.*  **end** *11. State update*

Line 1 is the state prediction stage of the standard CKF algorithm. From Line 2 to Line 10, the robustness factor is calculated for each received observation. The robustness factor, not only adjusts the UWB ranging error, but also distinguishes whether the positioning error is caused by the abnormal system state or the abnormal observation, so as to make a targeted adjustment. If the system state error and the observation error occur at the same time, the latter will be prioritized by the proposed algorithm and the system state error can only be corrected when a reliable observation is received. Line 11 is the state update of the standard CKF algorithm.

## 4. Experiments

**Figure 6:**
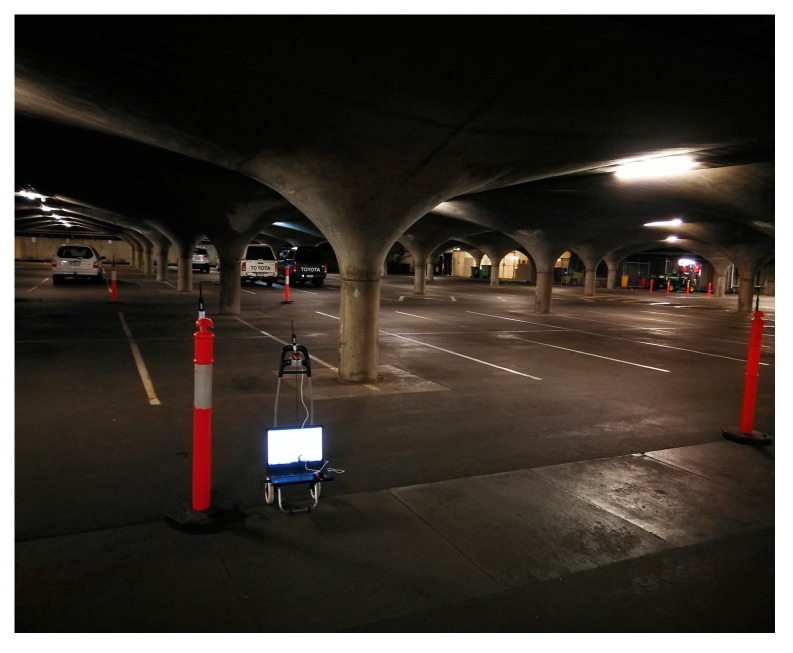
Test site in the underground garage.

**Figure 7:**
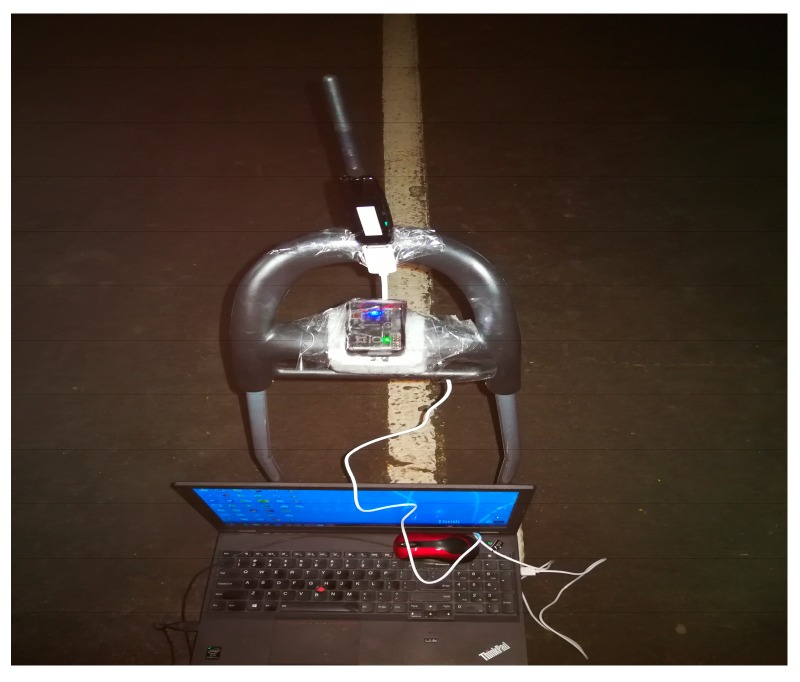
UWB tag and IMU.

For the evaluation of the proposed UWB/IMU fusion positioning method a test site was established in the underground garage of the University of Melbourne as shown in Figure 6. Four selected UWB beacons were placed on four brackets, forming a rectangular area of approximately 10 m × 5 m. The DWM1000 of Decawave Company (Burlingame, CA, USA) was adopted as the UWB tag/beacon, and was attached to a trolley. The X-IMU of the British company X-IO (London, UK) was selected as the IMU device, and was fixed 5 cm below the UWB tag, as shown in Figure 7. It is 55 × 35 × 18 mm (L × W × H) in size and almost 50 g in weight. Its host of on-board sensors, algorithms and configurable 8-channel auxiliary port make the x-IMU both a powerful sensor and controller. Communication is enabled via USB or Bluetooth for wireless applications. Its key technical specifications are shown in Table 1. The notebook on the trolley received the ranging data from the IMU and UWB at the same time, marking the same timestamp for the IMU data and UWB data. The trolley maintained a constant speed during its movement.

For IMU, the acceleration bias and the gyroscope bias were both dynamically estimated by CKF algorithm, and were used to correct observed values in real time. Axis misalignments error and scale factor error had already been corrected in the IMU calibration process. The other covariance parameters related to noise were set as follows in Table 2.

**Figure 8:**
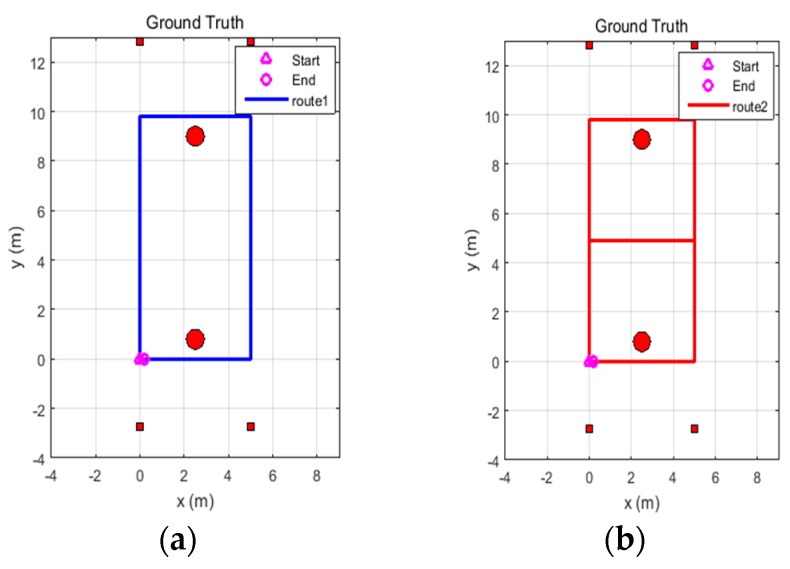
Map of the experiment setup and the reference trajectory. (**a**) Route 1; (**b**) Route 2.

Two routes were established for the experiments: Route 1 is a rectangular route with fewer turns and Route 2 is an 8-shaped route with more turns, as shown in Figure 8. Each route includes two laps, with both the starting point and the end point located in the lower left corner of the routes. The two red circles in the figure represent two large stone columns in the underground garage, and the rectangular red dots indicate the four beacons. The beacon coordinates and the ground truth trajectories were measured by a laser range finder. In the following, Route 1 is used for adaptivity analysis, and Route 2 is used for robustness analysis.

In the experimental analysis, the positioning result of the UWB, the UWB/IMU fusion algorithm based on standard CKF, and the proposed Adaptive-Robust CKF algorithm are denoted as UWB, CKF, and ARCKF, respectively.

### 4.1. Adaptivity Analysis

**Figure 9:**
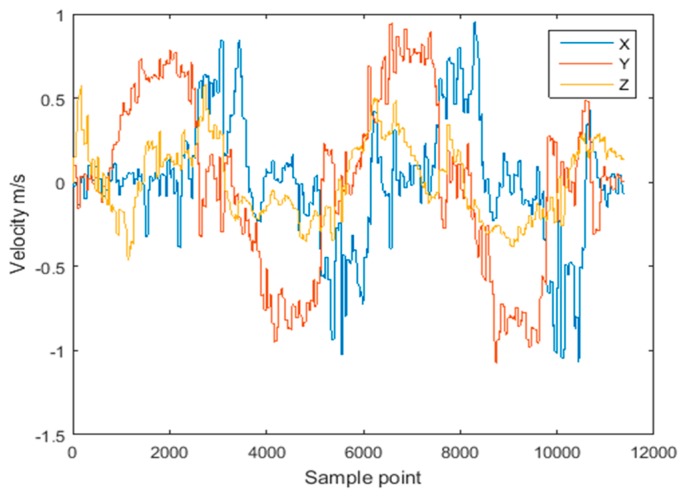
Velocity for Route 1.

**Figure 10:**
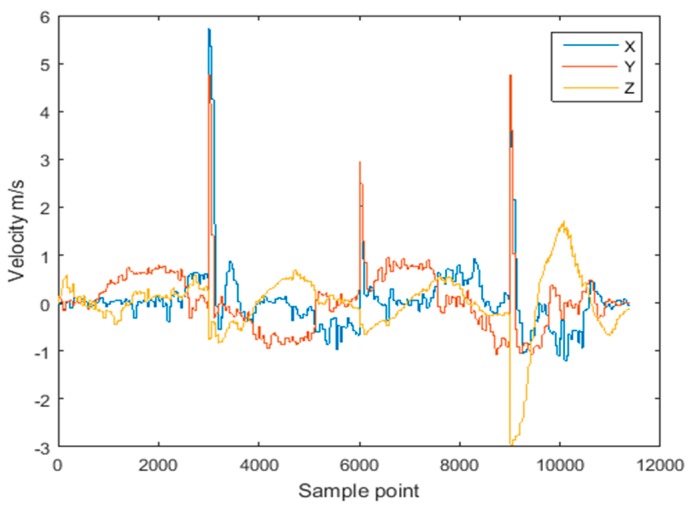
Velocity of inserting three noise for Route 1.

To simulate the effect of abnormal observations, the acceleration data recorded along Route 1 was contaminated with white Gaussian noise with an intensity of 50 dBW at three points, resulting in sudden changes of velocity. The comparison of velocity with and without the introduced abnormity is shown in Figure 9 and Figure 10, respectively. It can be seen that the sudden change of acceleration gives rise to the sudden change of velocity, further resulting in the abnormity of the estimated trajectory.

**Figure 11:**
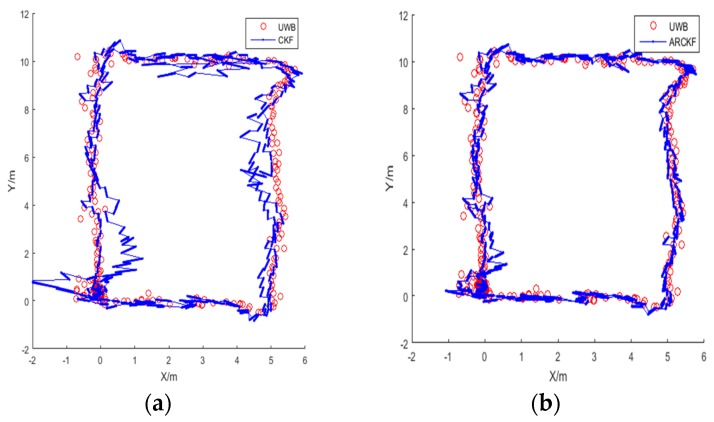
Positioning result for Route 1: (**a**) the standard CKF; (**b**) the proposed ARCKF.

Figure 11 shows the positioning result for Route 1. The red trajectory is the positioning result using the UWB data without any abnormity, and the blue trajectory is the positioning result of the UWB/IMU fusion with abnormal acceleration at 3 points.

Figure 11a shows the positioning result of the standard CKF algorithm, in which large deviations can be seen in the overall trajectory. Figure 11b shows the positioning result of the ARCKF algorithm. Although the trajectory estimated by the ARCKF contains some fluctuations, it is significantly improved as compared with the standard CKF.

In Figure 11b, the ARCKF algorithm can identify whether the positioning error is caused by the outlying system state or the outlying observation. Compared with the outlying observation, the system error caused by the outlying acceleration is more difficult to correct, because once the outlying system state occurs, the state error can be corrected only after the next observation is received. Thus, the accumulation of system errors during this period is inevitable. Furthermore, if the subsequent observation is still outlier, its suppression capability on system error is very limited, resulting in a continuously expanding error. This also explains the phenomenon seen in Figure 11b, that is, the influence of the outlying system state on the positioning result can only be suppressed to a certain extent, but cannot be completely eliminated.

**Figure 12:**
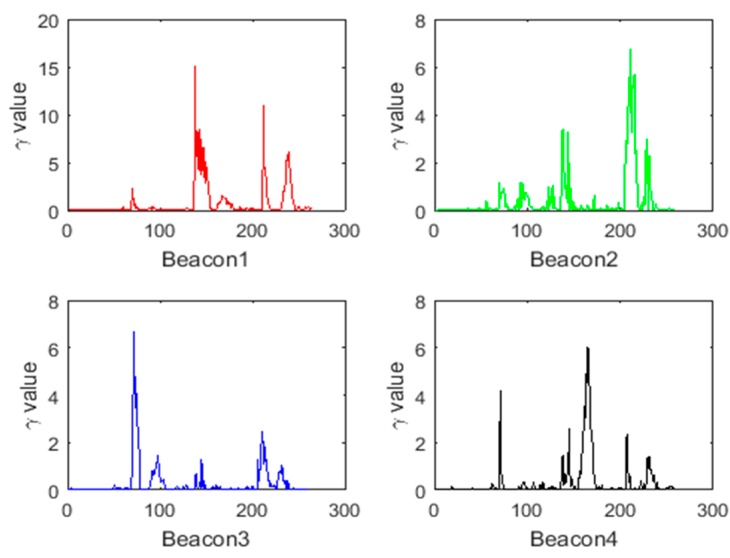
The values of $\gamma_{k}$ for the four beacons computed along Route 1.

Figure 12 shows the values of $\gamma_{k}$ for the four beacons computed along Route 1. As it can be seen, values that are greater than the threshold are present in the $\gamma_{k}$ corresponding to the four beacons, indicating an abnormal system state. The covariance of the system state is adjusted by $\gamma_{k}$, and the influence of the abnormal system state on the positioning result is reduced, so that the positioning result is closer to the observation result. Additionally, after receiving the correct observations, the backward RTS smoothing can be applied until the data point which makes the system state abnormal, smoothing the system cumulative error during this period.

**Figure 13:**
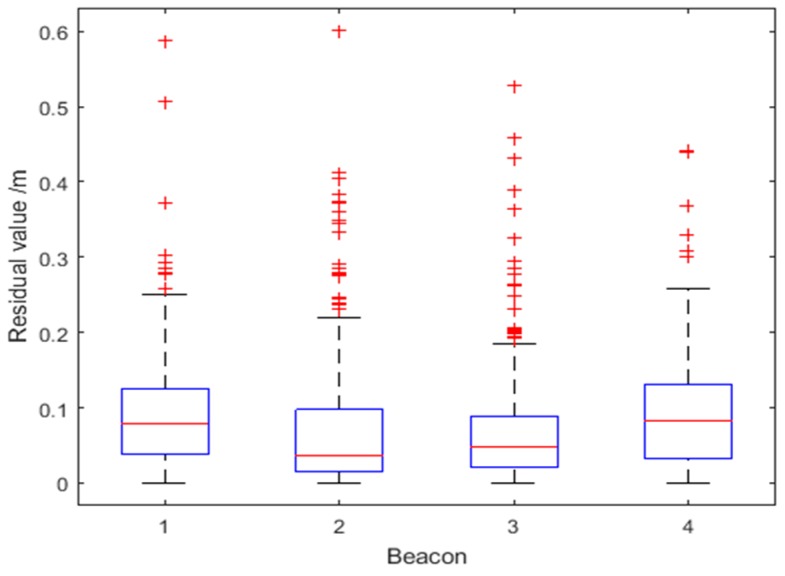
Distributions of residual values for the four Beacons for Route 1.

Figure 13 shows the residual values $\Delta r_{i}$ corresponding to the four UWB beacons for Route 1. The optimized residual values are unevenly distributed, but on the whole they have a relatively small range, with a maximum optimized residual of about 0.6 m. There are two reasons for the uneven distribution of residuals: first, the values at some time points jump when the ranging value is blocked by the column or interfered by the pedestrian; second, with the increase of the distance, the UWB ranging error will also increase relatively, and the corresponding residuals will increase accordingly. By analyzing the proportion of the optimized residuals, the robustness factor is dynamically determined to obtain the weight of the observation covariance for all beacons.

**Figure 14:**
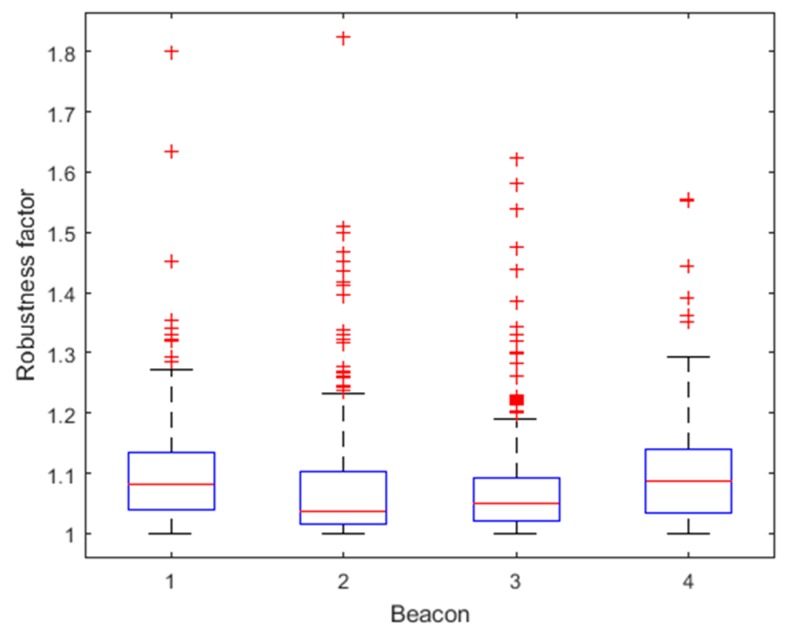
Distributions of robustness factors for the four Beacons for Route 1.

Figure 14 shows the distribution of robustness factors calculated from residuals $\Delta r_{i}$. Table 3 lists the number of times the robustness factor reaches the thresholds for each beacon. It can be seen that most data are below $C_{u\_max}$, with only one time reaching the threshold at Beacon 1 and 2, respectively, indicating that the data are basically reliable. The larger the $\Delta r_{i}$, the larger the corresponding robustness factor is, so that the observation covariance can be dynamically increased to reduce the influence of the ranging error on the fusion algorithm, improving the accuracy and robustness of the fusion algorithm.

Table 4 illustrates the root mean square (RMS) positioning error of the proposed ARCKF algorithm compared to the standard CKF when artificial outliers are introduced in the acceleration data. The positioning accuracy of the CKF algorithm decreases rapidly with the increase of the number of outliers; the ARCKF algorithm is also affected by the outliers, however its error is only about half of the error of the CKF algorithm, demonstrating good adaptability to outlying measurements.

### 4.2. Robustness Analysis

**Figure 15:**
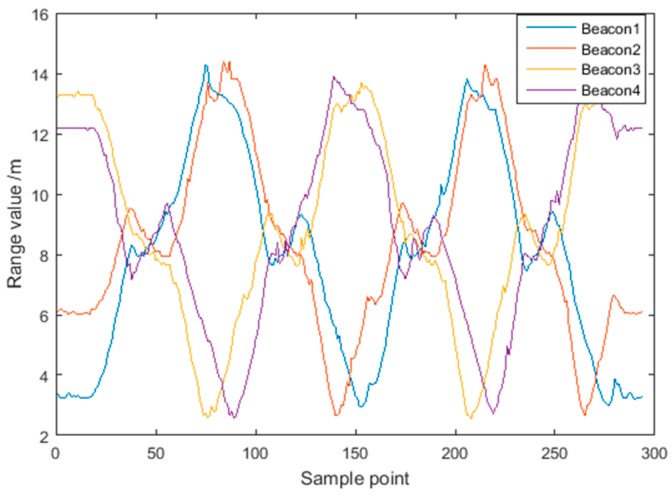
UWB range values for Route 2.

To evaluate robustness, the UWB data recorded along Route 2 were contaminated with white Gaussian noise with an intensity of 20 dBW for Beacon 2 and Beacon 4. The noisy data account for 10% of the total distance measurements. Figure 15 shows the distance measurements for the four UWB beacons without noise.

**Figure 16:**
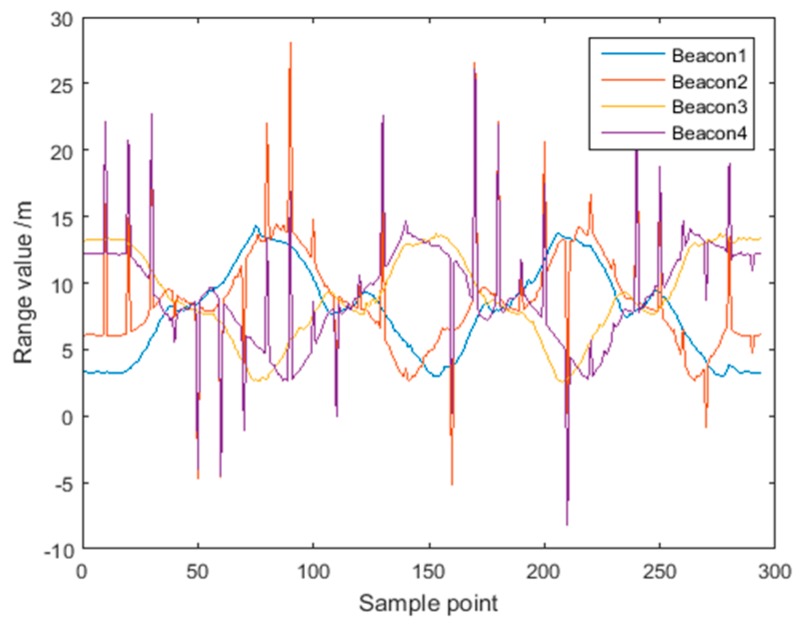
UWB range values for Route 2 where 10% of data from Beacons 2 and 4 are contaminated with noise.

**Figure 17:**
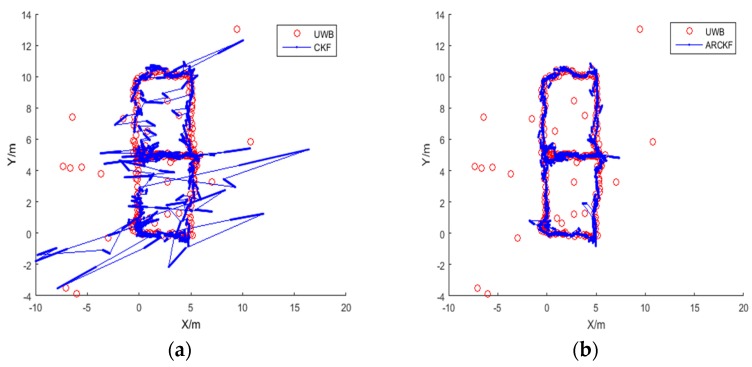
Positioning result for Route 2: (**a**) the standard CKF; (**b**) the proposed ARCKF.

Figure 16 shows the distance measurements for the four UWB beacons with noise. The positioning results for Route 2 are shown in Figure 17. The red trajectory is the positioning result of the UWB, where 10% of data from Beacon 2 and Beacon 4 contain white Gaussian noise randomly added. The blue trajectory is the positioning result of fusing original acceleration data and UWB data. As shown in Figure 17a, the positioning accuracy of standard CKF is severely disturbed by the observation noise, and many points with large deviations from the reference trajectory can be seen. This is because the CKF algorithm sets a fixed covariance for the measurements, which is used to estimate the maximum posterior distribution of the system state. Thus, abnormal UWB measurements have a serious impact to the estimation of the posterior distribution.

In comparison, the accuracy of the ARCKF algorithm shown in Figure 17b is much higher. Most of the deviations caused by abnormal UWB observations are eliminated, presenting a smoother trajectory. This is because when the residual of an observation is very large, that is, $C_{ui}\geq C_{u\_max}$, the covariance of this observation is amplified by the method based on Mahalanobis distance, thereby reducing its effect on the system state. When $C_{ui}<C_{u\_max}$, the observation error is adjusted by the robustness factor to improve the overall positioning accuracy.

**Figure 18:**
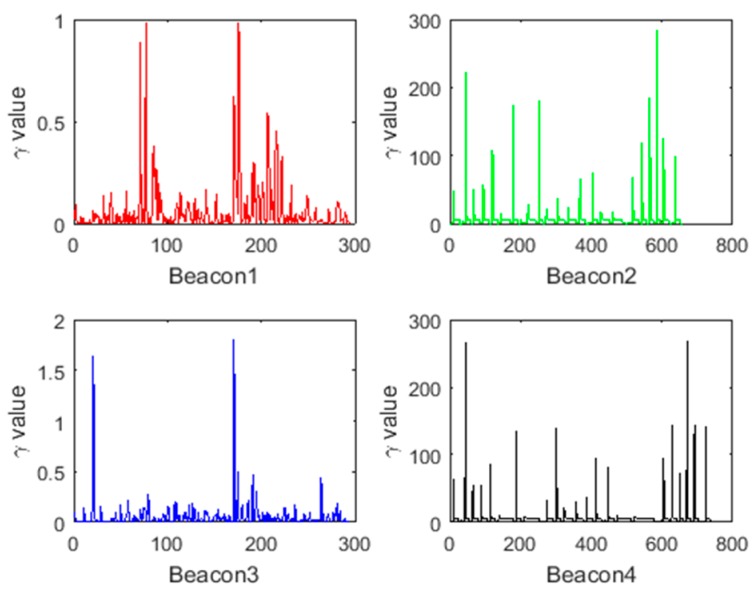
The values of $\gamma_{k}$ for the four beacons computed alongRoute 2.

Figure 18 shows the values of $\gamma_{k}$ for the four beacons computed along Route 2. As it can be seen, for Beacon 2 and Beacon 4, there are multiple values that are much larger than the threshold of 6.2, indicating that there are abnormal observations; whereas the $\gamma_{k}$ for Beacon 1 and Beacon 3 are all less than 6.2, which is consistent with the experiment as these two beacons did not contain any abnormal values.

**Figure 19:**
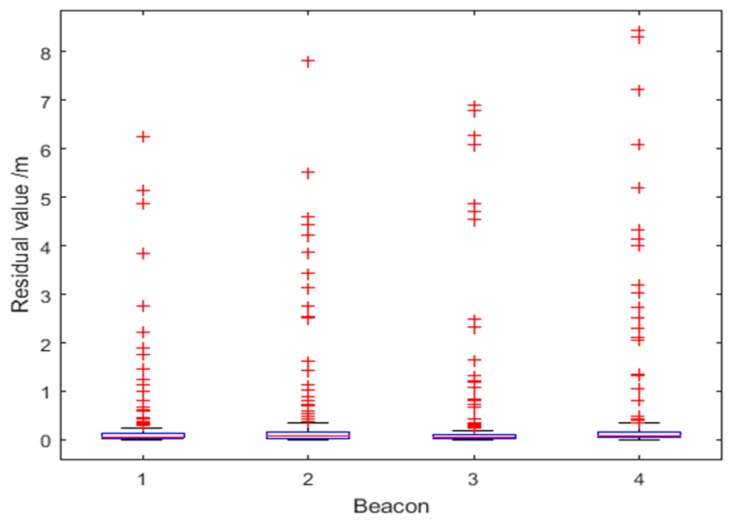
Distributions of residual value for the four Beacons for Route 2.

Figure 19 shows the residual values $\Delta r_{i}$ corresponding to the four UWB beacons for Route 2. Since the data of Beacon 2 and Beacon 4 contain outliers, the optimized residuals are significantly larger with a maximum optimized residual of about 8 m. Although the data of Beacon 1 and Beacon 3 are not contaminated with noise, their optimized residuals are correspondingly increased due to the influence of the data of Beacon 2 and Beacon 4.

**Figure 20:**
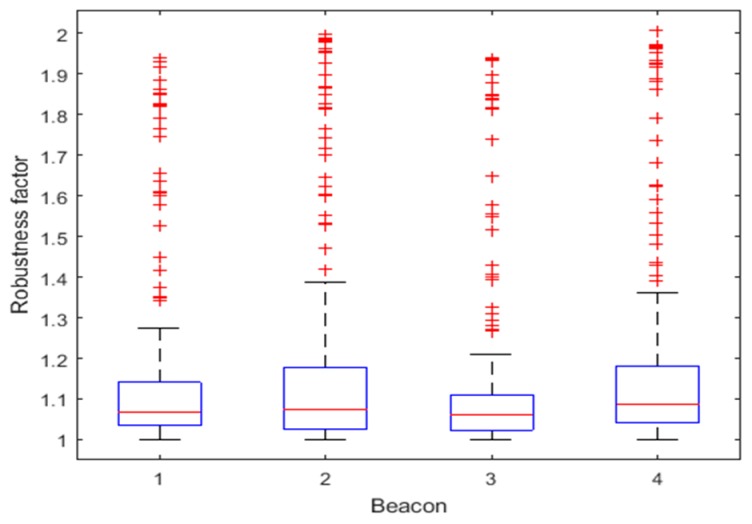
Distributions of robustness factors for the four Beacons for Route 2.

As shown in Figure 20 and Table 5, the robustness factor of the four beacons reaches the threshold $C_{u\_max}$ multiple times, indicating the presence of abnormal ranging data. The proposed method based on Mahalanobis distance can dynamically increase the observation covariance, reducing the impact of these abnormal ranging data on the fusion algorithm.

Table 6 illustrates the root mean square (RMS) positioning error of the proposed ARCKF as compared to standard CKF when artificial outliers are introduced in the UWB range measurements. With the increasing percentage of added noise, the positioning accuracy of the CKF algorithm decreases rapidly, whereas the accuracy of the ARCKF algorithm is relatively stable. When 10% of ranging data is noisy, the ARCKF algorithm still maintains a positioning accuracy of 0.59 m, demonstrating robustness to the added noise.

The lower bounds of positioning error is estimated by CRLB (Cramer-Rao Lower Bounds). In the application of Bayesian filter, the method based on iteration is usually adopted to estimate the posterior CRLB, so as to update the CRLB in each step. The CRLB at Moment *k* could be defined as:

(43) $$CRLB=J_{k}^{-1}$$

where, $J_{k}$ represented the Fisher information matrix of system state $X_{k}$.According to [23], the Fisher information matrix $J_{k+1}$ could be calculated based on the Fisher information Matrix at Moment k, the system transform matrix $F_{k+1}$ and the measurement matrix $H_{k+1}$.

**Figure 21:**
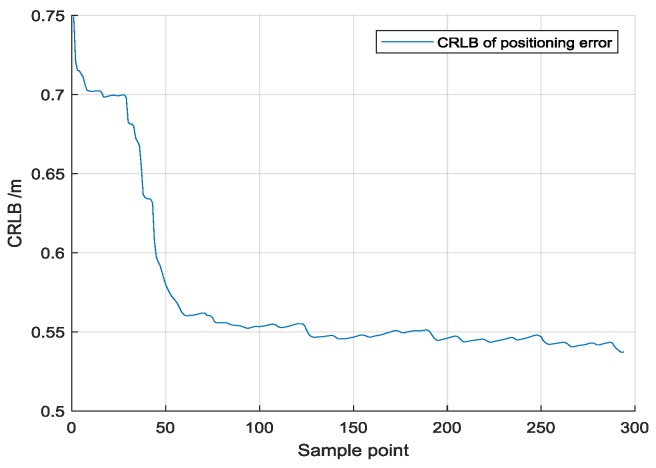
CRLB of positioning error for Route 2.

It can be shown that:

(44) $$J_{k}=H_{k+1}^{T}R_{k+1}^{-1}H_{k+1}+{\left(Q_{k}+F_{k}J_{k}^{-1}F_{k}^{T}\right)}^{-1}$$

where $Q_{k}\;\mathrm{and}\;R_{k+1}$ are represented the covariance matrix of state transaction function at Moment *k* and the covariance matrix of measurements at Moment *k* + 1. For Route 2, when injected with 10% UWB ranging noise, the calculated CRLB is shown in Figure 21.

### 4.3. Positioning Result of Different Number of Beacons

**Figure 22:**
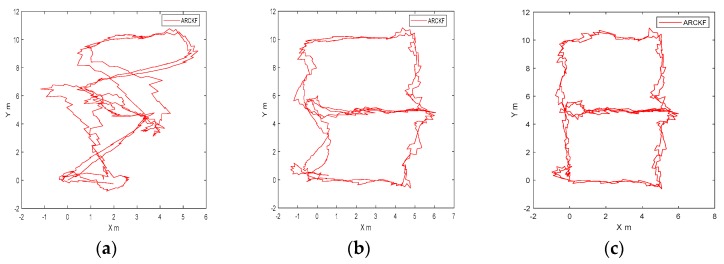
Positioning result of different numbers of beacons for Route 2: (**a**) 2 beacons; (**b**) 3 beacons; (**c**) 4 beacons.

In order to verify the positioning accuracy of the fusion algorithm under different numbers of beacons, the positioning trajectories of 2, 3 and 4 beacons were given, as shown in Figure 22a–c, respectively. Figure 22a shows that the trajectory was seriously distorted. Since the accumulative error of IMU integral increased rapidly along with the time, the distance measurement value from only 2 beacons at the left diagonal line could not restrain the whole trajectory effectively. Figure 22b shows that under the restraint of 3 beacons, the positioning accuracy was better than that of 2 beacons, but there were still some deviations in the trajectory of Y-axis. Figure 22c shows that under the restraint of 4 beacons, the trajectory was roughly the same to the reference trajectory. Theoretically, the more the beacons, the stronger the restraint of IMU integral results would be. However, in actual application, the positioning area covered by four beacons could satisfy the requirement of positioning accuracy to some extent.

## 5. Conclusions

In this paper, a UWB/IMU fusion method for indoor positioning based on Adaptive-Robust CKF is presented. The Mahalanobis distance between the observation and the system state is calculated in the algorithm to update the covariance of the observation or system state, thereby reducing the effect of the abnormal observations or system state on the positioning result. In addition, a method for calculating the robustness factor when the observation error is smaller than a threshold is proposed in this paper, which guides the algorithm to appropriately apply robust filtering or adaptive-robust filtering. The experimental results show that the proposed method presents a strong error recovery capability. When affected by abnormal data, it can achieve a positioning accuracy much higher than that of the standard CKF algorithm. Future improvements could include the following: the trolley used in our experiments moved relatively stably. For more complex movement patterns, such as rapid acceleration and emergency stop, a better motion state model is required to fit the tested movement pattern. In addition, fusion with other types of data such as geomagnetic data could further improve the positioning accuracy and deserves further investigation.

## Figures and Tables

**Table 1:** Key specifications of the IMU.

Instrument	Sensitivity	Zero-Point Offset	Noise Density	Max Range
Accelerometer	0.9~4.3 mg/bit	±50 mg	218 μg/√HZ	±8 g
Gyroscope	32.8~131 LSB/(°/s)	±20°/s	0.01°/s/√HZ	±2000°/s
Magnetometer	0.3 μT/LSB	N/A	N/A	±8 G

**Table 2:** Parameter list of IMU noise.

Name	Symbol	Value
Accelerometer noise covariance	$N_{a}$	(0.1 g)^2^
Gyroscope noise covariance	$N_{g}$	(0.2 rad/s)^2^
Accel bias drift noise covariance	$U_{a}$	(0.01 g)^2^
Gyro bias drift noise covariance	$U_{g}$	(0.02 rad/s)^2^

**Table 3:** The number of times the robustness factor reaches the threshold $C_{u\_max}$.

Route 1 RMSE (m)				
Beacon	1	2	3	4
times	1	1	0	0

**Table 4:** RMSE of positioning with artificial outliers (m).

Route 1 RMSE (m)				
Number of injecting noise	1	2	3	4
CKF	0.87	1.26	1.56	2.27
ARCKF	0.49	0.58	0.79	1.02

**Table 5:** The number of times the robustness factor reaches the threshold $C_{u\_max}$.

Route 2 RMSE (m)				
Beacon	1	2	3	4
times	18	23	18	22

**Table 6:** RMSE of positioning with noise added to UWB range measurements.

Route 2 RMSE (m)				
Percentage of added noise	3%	5%	7%	10%
CKF	0.67	1.06	1.36	1.67
ARCKF	0.39	0.45	0.52	0.59
